# Malaria Outbreak in Farafangana District, Southeast Madagascar, 2018: Are Secondary Vectors a Threat to Current Malaria Control Approaches?

**DOI:** 10.4269/ajtmh.24-0834

**Published:** 2025-10-23

**Authors:** Thiery Nepomichene, Aina Harimanana, Fenomiaranjara Randrianaivo, Rico Randrenjarison, Rogelin Raherinjafy, Jean Marius Rakotondramanga, Sarah Zohdy, Solofo Razakamiadana, Laurent Kapesa, Mauricette Nambinisoa Andriamananjara, Laurence Baril, Rindra Randremanana, Laurence Randrianasolo, Catherine M. Dentinger, Romain Girod

**Affiliations:** ^1^ Institut Pasteur de Madagascar, Antananarivo, Madagascar;; ^2^ Ministry of Public Health, National Malaria Control Program, Antananarivo, Madagascar;; ^3^ Division of Parasitic Diseases and Malaria, US Centers for Disease Control and Prevention, Atlanta, Georgia;; ^4^ US President’s Malaria Initiative, Antananarivo, Madagascar

## Abstract

A malaria outbreak occurred in Farafangana District, Madagascar, in 2018, shortly after the implementation of insecticide-treated bed net distribution and indoor residual spraying campaigns. Entomological and epidemiological investigations were conducted to characterize disease transmission in six villages in three communes. Mosquitoes were collected using human landing catches, light traps, and pyrethrum spray catches. Vector biting behavior was described, and sporozoite indices were determined. To describe demographic and risk data, questionnaires were administered to individuals from randomly selected households, and rapid diagnostic tests (RDTs) were performed on consenting household members. *Anopheles coustani* (*An. coustani*), *Anopheles gambiae* s.s. (*An. gambiae* s.s.), *Anopheles funestus* (*An. funestus*), and *Anopheles mascarensis* (*An. mascarensis*) were the most frequently captured malaria vector species. Outdoor biting was common for all predominant *Anopheles* species collected (exophagy rates varied from 59.8% for *An. gambiae* s.s. to 100.0% for *An. coustani*), except for *An. funestus*, which exhibited an exophagy rate of less than 47.0%. Of 1,488 *Anopheles* mosquitoes collected, 25 (1.7%) had *Plasmodium falciparum* sporozoites, only one of which was collected indoors. The remaining 24 were collected outdoors, 13 (54.2%) of which were *An. coustani*. The other 12 specimens were *An. funestus*, *Anopheles squamosus*, *An. gambiae* s.s., and *An. mascarensis*. Of 226 individuals tested using RDTs, 71 (31.4%) had positive results. A total of 61 (85.9%) of these individuals were asymptomatic, most of whom were children. Highly infected secondary malaria vectors, in addition to primary vectors, combined with a predominance of exophagy, contributed to parasite transmission in the Farafangana District, where indoor-targeted vector control measures had been implemented. A high proportion of asymptomatic infections likely sustained transmission. Control strategies for outdoor biting should be explored.

## INTRODUCTION

Malaria is endemic in Madagascar, and although the entire population is at risk for the disease, transmission is heterogeneous across the island nation, with focal disease clusters occurring periodically.^1^^,^^2^ The Atsimo-Atsinanana Region on the southeastern coast has one of the highest malaria rates in the country. The Farafangana District, located in this region, had an average annual incidence of 68.6 cases per 1,000 inhabitants from 2010 to 2019. Malaria transmission varies seasonally, increasing during the rainy season, from October to April.^3^^,^^4^ Geographic and climatic characteristics of the district, including low elevation (0–50 m), tropical temperatures (an average of 22°C, with seasonal variations ranging from 15°C in July to 29°C in February), and heavy rainfall (>2,500 mm annually), may facilitate *Anopheles* mosquito breeding and *Plasmodium* transmission.^5^ In addition, Farafangana is a rural, primarily agricultural district, which contributes to the persistence of malaria. Agricultural practices, such as converting forests to rice paddies or raising livestock, may facilitate the proliferation of vectors and the maintenance of parasites.^6^^–^^9^

In 2012, an investigation was conducted that included a cross-sectional survey of 1,615 individuals from 440 households in two southeastern regions of Madagascar, including Atsimo-Atsinanana, in response to an increase in reported malaria cases. The survey revealed a higher risk of malaria among individuals older than 6 months of age, residents of rural settings, and those in the lowest socioeconomic tercile. Infection rates were lower among those who reported regularly sleeping under an insecticide-treated bed net (ITN). The investigation also revealed reported stockouts of anti-malarial drugs in the previous 6 months in two-thirds of the health facilities surveyed. Additionally, cumulative rainfall was higher that year compared with the 3 previous years.^2^ To describe vector behavior, entomological monitoring was conducted bimonthly from 2014 to 2017 in three villages in the Farafangana District. This monitoring revealed that *Anopheles funestus* (*An. funestus*), *Anopheles coustani* (*An. coustani*), and *Anopheles gambiae* s.s. (*An. gambiae* s.s.) were the predominant species, and that human biting rates (HBRs) were higher outdoors than indoors for all species except *An. funestus.* Moreover, the annual entomological inoculation rates (EIRs) in 2014 were higher for *An. funestus* (39.4 infectious bites per human [ibph]) than for *An. gambiae* s.s. (24.3 ibph). In 2017, the highest EIR was observed for *An. coustani*, with 11.3 ibph. When considering all 4 years, the monthly EIR for *An. funestus* was highest in May, indicating that transmission due to this predominant species was higher at the end of the rainy season (Institut Pasteur de Madagascar [IPM], unpublished data).

To address the identified risks for malaria in the Atsimo-Atsinanana Region, Madagascar’s National Malaria Control Program (NMCP) and its partners initiated indoor residual spraying (IRS) and ITN distribution campaigns. Beginning in 2015, IRS was offered to all households in the Farafangana District annually through 2017 using Actellic^®^ 300CS, a pirimiphos-methyl (organophosphate)-based insecticide. In August 2018, IRS was conducted using SumiShield^®^ 50WG, a clothianidin (neonicotinoid)-based insecticide, with an overall coverage of 97.5% of identified and accessible structures.^10^ In addition, ITNs were distributed throughout the district in 2015 and 2018 (DawaPlus^®^ 2.0) as part of nationwide campaigns. In 2017, a program of continuous ITN distribution was initiated to ensure high ITN coverage between mass distribution campaigns. In September 2018, one DawaPlus 2.0 net was distributed for every two persons in the district (Population Services International Madagascar, unpublished data). These indoor residual spraying and ITN distribution initiatives were reinforced through communication campaigns. In addition, efforts to ensure high-quality case management for children under 5 years of age by community health volunteers (CHVs) in villages, as well as case management for all ages at the commune level (through health facilities), were reinforced. This included delivering adequate diagnostic tools (malaria rapid diagnostic tests [RDTs]) and proper treatment supplies (e.g., artemisinin-based combination therapy [ACT]).

In November 2018, shortly after the IRS and ITN distribution campaigns were completed, Farafangana health authorities detected an increase in malaria cases that exceeded their epidemiological threshold during the epidemiological week 47 (November 19–25). Health facilities in three of the 33 communes, including Ankarana, Iabohazo, and Tovona, were affected. The NMCP initiated a rapid response starting January 30, 2019, in nine villages in the three communes. The NMCP conducted active case detection, and all residents of these villages were encouraged to be tested for malaria via RDT from January 31, 2019 to February 13, 2019. Of 1,361 residents tested (∼5% of the population), 401 (29.5%) were positive. The highest positivity rate (48.0%) was observed among children 6–14 years of age. Anecdotal reports of commodity stockouts, security concerns, and road washouts likely prevented residents from obtaining or seeking care. Additional anecdotal information that might have contributed to the increase included houses that had not received IRS during the campaign, outdoor living that extended until late at night, and a preference for seeking care from traditional healers over trained healthcare workers (NMCP, unpublished data).

To further investigate the 2018 malaria outbreak in the Farafangana District, an entomological investigation, as well as household and individual surveys, were conducted to explore the risks associated with malaria in the three affected communes. The findings from these surveys are reported in the present study, and their implications are discussed.

## MATERIALS AND METHODS

### Study setting and design.

**Figure 1.**
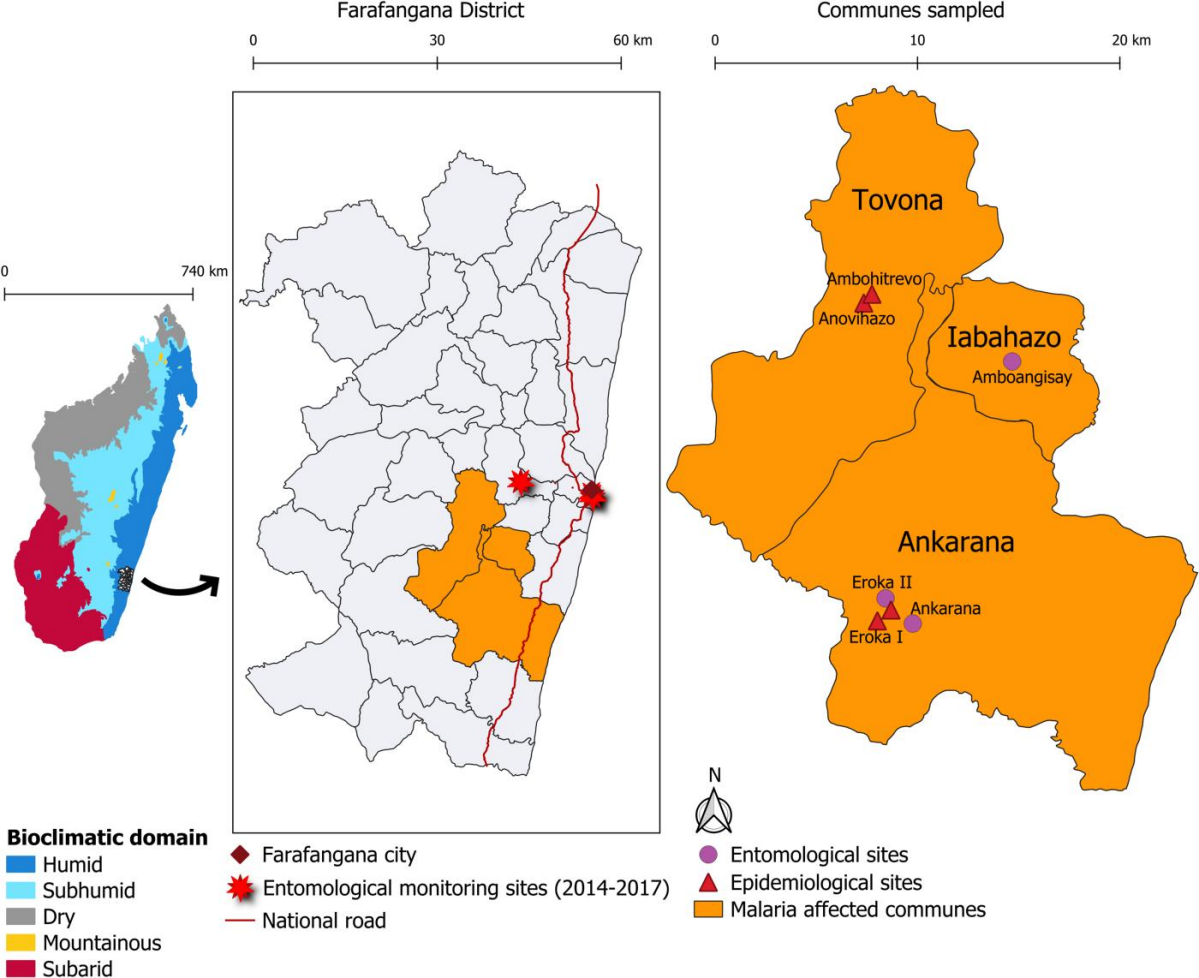
Entomological and epidemiological investigation sites, Farafangana District, Madagascar, February and April 2019.

The investigation was conducted in six villages in the communes of Tovona, Iabohazo, and Ankarana. The entomological investigation took place from February 28, 2019 to March 13, 2019 in the three villages of Amboangisay (Iabohazo Commune) and Ankarana and Eroka II (Ankarana Commune). The household and individual surveys, including testing via RDT, were conducted from April 6, 2019 to April 14, 2019, in the four villages of Ambohitrevo and Anovihazo (Tovona Commune) and Eroka I and Eroka II (Ankarana Commune; Figure 1).

### Entomological investigation.

#### Mosquito collection.

Mosquitoes were collected using human landing catches (HLCs), pyrethrum spray catches (PSCs), and CDC light traps (LTs). Human landing catches were performed over three consecutive nights, from 6:00 pm to 6:00 am, inside and outside three houses in each of the villages of Amboangisay and Ankarana. Mosquitoes were detected using a flashlight and collected with glass tubes before biting occurred, and they were packaged in collecting bags hourly. Collectors switched places each night to avoid sampling bias related to variability in mosquito attraction to the human hosts. In each of the same two villages, PSCs were performed once: one morning from 6:30 to 8:30 am in five randomly selected houses where HLCs had not occurred. The doors and windows of each house were closed, and the eaves were covered to prevent mosquitoes from escaping. The floors and furniture were covered with drop cloths to catch any fallen mosquitoes. Fifteen minutes after spraying the house walls with pyrethroid insecticide, dead and paralyzed mosquitoes were collected. CDC LTs were set up once to capture mosquitoes: one night between 6:00 pm and 6:00 am, outside, but close to, three randomly selected houses in the villages of Amboangisay and Eroka II.

#### Mosquito identification and laboratory processing.

Mosquito identification was performed in the field using a binocular loupe, according to Madagascar-specific morphological keys.^8^ At the IPM, DNA extracted from the legs or wings of mosquitoes morphologically identified as *An. gambiae* s.l. was used for polymerase chain reaction (PCR) species identification, using specific primers to discriminate among *An. gambiae* s.s., *Anopheles arabiensis* (*An. arabiensis*), and *Anopheles merus*, as described by Scott et al.^11^ The detection of antigens of the infectious human *Plasmodium* parasites, sporozoites, in mosquitoes was conducted on homogenized heads and thoraces of *Anopheles* females using circumsporozoite (CSP) ELISA. Both *Plasmodium falciparum* (*P. falciparum*) and *Plasmodium vivax* (*P. vivax*; PV 210 and PV 247) CSP antigens were investigated according to the protocol developed by malaria research and reference reagent resource center.^12^ All samples that tested positive for *P. falciparum* or *P. vivax* were confirmed by performing quantitative PCR (qPCR) testing on DNA extracted from head and thorax lysates, as described by Canier et al.^13^

#### Household and individual surveys.

Households were randomly selected for participation, a head of household was identified, and consent was obtained to complete the household survey. This survey included information on sociodemographic characteristics; distance from cattle pens; the presence, use, and care of ITNs; and whether the house received IRS. The individual survey included information regarding fever illness, care-seeking behavior, and knowledge and perceptions of ITN use and care (Supplemental Table 1). Children <18 years of age responded if they were able; otherwise, the parent or guardian responded on behalf of the child.

### Laboratory analysis.

Malaria AG Pf/*Pan* rapid diagnostic tests (SD Bioline, Memphis, TN, Lot no. 05EDD052A) were performed on each consenting individual according to the manufacturer’s instructions.^14^ Thick and thin smears were field-prepared and analyzed at IPM in accordance with WHO guidelines. Slides were read by two trained microscopists. The reported parasite density was the mean value of the densities estimated by each of the two microscopists. However, for density discrepancies >25%, a third microscopist read the slide, and the mean value from all three microscopists was reported.

## STATISTICAL ANALYSES

Entomological parameters were measured for each *Anopheles* species in each village. The relative abundance was calculated on the basis of the total number of *Anopheles* mosquitoes collected, and the HBR was calculated on the basis of HLC collections and expressed as the number of bites per human per night (BHN). Hourly HBRs were calculated to determine the nocturnal biting behavior of each anopheline species. Indoor and outdoor HBRs were calculated to estimate exo- and endophagic trends. The sporozoite index was estimated as the proportion of mosquitoes positive for *Plasmodium* spp. among the total number of mosquitoes tested.

Epidemiological survey and RDT results were recorded in the field on paper and were double-entered into REDCap 8.5.17 (Vanderbilt University, Nashville, TN) at IPM.^15^^,^^16^ Database cleaning and statistical analyses were performed using STATA 17.0 2021 (Stata Corporation, College Station, TX). Continuous variables were expressed as medians with interquartile ranges (IQRs). Qualitative variables were expressed as absolute numbers and percentages. Household-level group data (e.g., proximity to cattle pens and IRS exposure) were analyzed by household and assigned as a characteristic for individuals in that household. A generalized linear model (binomial) was used to assess the association between RDT-positivity and age, sex, fever or recent fever, distance from cattle pens, ITN use, and IRS coverage. The level of significance was set at 0.05.

## RESULTS

### Entomological investigation.

#### *Anopheles* composition and species abundance.

A total of 2,176 anopheline mosquitoes were collected. Of these, 1,488 (68.4%) were captured via HLC. At least 12 *Anopheles* species were identified: 11 in Amboangisay (HLC, PSC, CDC LT), eight in Ankarana (HLC, PSC), and five in Eroka II (CDC LT).

In Amboangisay, among the 306 *An. gambiae* s.l. tested via PCR, four (1.3%) were identified as *An. arabiensis*, and the remaining were identified as *An. gambiae* s.s. *Anopheles coustani* was predominant (*n* = 475; 35.2%), followed by *An. funestus* (*n* = 359; 26.6%), *An. gambiae* s.s (*n* = 293; 21.7%), and *Anopheles squamosus* (*An. squamosus*; *n* = 124; 9.2%). Among the 252 anopheline mosquitoes captured using CDC LTs placed outdoors, *An. squamosus* was the predominant species (*n* = 137; 54.4%), followed by *An. coustani*.

In Ankarana, HLC was used to capture 140 anopheline mosquitoes, with *An. gambiae* s.s. being predominant (*n* = 62; 62.1%), followed by *An. funestus* (*n* = 27; 19.3%) and *Anopheles mascarensis* (*An. mascarensis*; *n* = 14; 10.0%). No anopheline mosquitoes were captured using PSC.

In Eroka II, 433 anopheline mosquitoes were collected using CDC LTs, most of which were *An. squamosus* (*n* = 313; 72.3%). All *An. gambiae* s.l. mosquitoes were identified as *An. gambiae* s.s. via PCR testing (Table 1).

**Table 1:** Relative abundance of anopheline species by village and collection method, Amboangisay, Ankarana, and Eroka II villages, Farafangana District, Madagascar, February 2019

Species	Amboangisay			Ankarana	Eroka II
	HLC (%)	PSC (%)	CDC LT (%)	HLC (%)	CDC LT (%)
*Anopheles coustani*	475 (35.2)	0 (0.0)	94 (37.3)	5 (3.6)	30 (6.9)
*Anopheles squamosus*	124 (9.2)	0 (0.0)	137 (54.4)	1 (0.7)	313 (72.3)
*Anopheles gambiae* s.s.	293 (21.7)	1 (33.3)	8 (3.2)	87 (62.1)	88 (20.3)
*Anopheles funestus*	359 (26.6)	2 (66.7)	11 (4.4)	27 (19.3)	1 (0.2)
*Anopheles mascarensis*	20 (1.5)	0 (0.0)	0 (0.0)	14 (10.0)	0 (0.0)
*Anopheles pauliani*	25 (1.9)	0 (0.0)	0 (0.0)	0 (0.0)	0 (0.0)
*Anopheles pretoriensis*	23 (1.7)	0 (0.0)	0 (0.0)	2 (1.4)	0 (0.0)
*Anopheles flavicosta*	21 (1.6)	0 (0.0)	0 (0.0)	2 (1.4)	0 (0.0)
*Anopheles arabiensis*	4 (0.3)	0 (0.0)	0 (0.0)	0 (0.0)	0 (0.0)
*Anopheles maculipalpis*	3 (0.2)	0 (0.0)	0 (0.0)	0 (0.0)	0 (0.0)
*Anopheles pharoensis*	0 (0.0)	0 (0.0)	0 (0.0)	2 (1.4)	1 (0.2)
*Anopheles brunnipes*	0 (0.0)	0 (0.0)	2 (0.8)	0 (0.0)	0 (0.0)
*Anopheles* sp.	1 (0.1)	0 (0.0)	0 (0.0)	0 (0.0)	0 (0.0)
Total	1,348 (100.0)	3 (100.0)	252 (100.0)	140 (100.0)	433 (100.0)

HLC = human landing catch; LT = light trap; PSC = pyrethrum spray catch.

#### Trophic behavior of malaria vectors.

**Figure 2.**
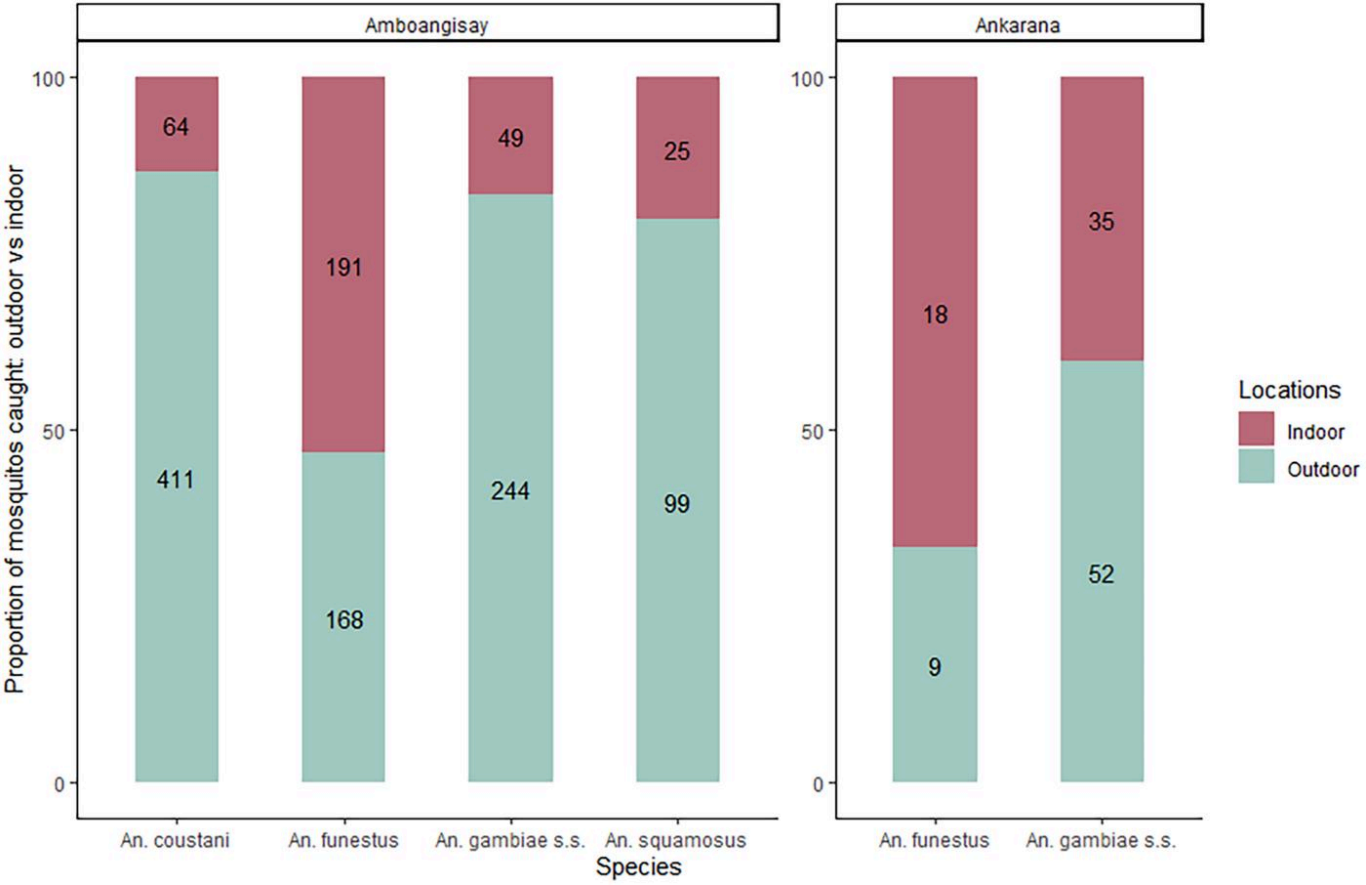
Proportion of outdoor and indoor bites of predominant anopheline mosquito species in Amboangisay and Ankarana, Farafangana District, Madagascar, February 2019. Only *Anopheles funestus* exhibited endophagic behavior, with more than 50% of bites occurring indoors.

In Amboangisay, all predominant species exhibited evident exophagy except for *An. funestus*. For *An. coustani*, *An. gambiae* s.s., and *An. squamosus*, more than 80.0% of bites were observed outdoors. Exophagy for *An. funestus* was found among fewer than 50.0% of HLCs. In Ankarana, *An. funestus* exhibited endophagy for more than 65.0% of HLCs, whereas *An. gambiae* s.s. was predominantly exophagous, with more than 58.0% of bites observed outdoors (Figure 2).

#### Hourly variations in HBRs.

**Figure 3.**
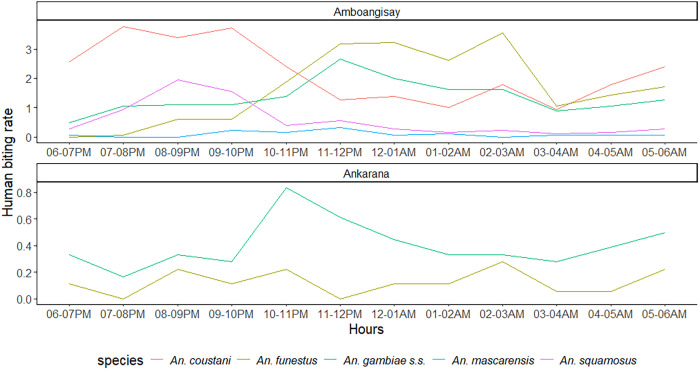
Hourly human biting rates of predominant anopheline mosquito species, Amboangisay and Ankarana, Farafangana District, February 2019. For the two sites, based on the human biting rate, mosquito bites likely start before 6 pm and continue after 6 am; however, measurements were only recorded from 6 pm to 6 am.

In Amboangisay, the HBR of the predominant species, *An. coustani*, was 26.4 BHN. Peak aggressiveness was observed between 7 and 10 pm, with 3.8 bites per human per hour (BHH); 24.0% of bites occurred from 6 to 8 pm, when villagers are often outside their homes and unprotected. The HBR of *An. funestus* was 19.9 BHN; it increased from 10 pm to 3 am, when 72.4% of bites were observed. Peak aggressiveness was observed from 2 to 3 am, with 3.5 BHH. The HBR of *An. gambiae* s.s. was 16.3 BHN, and bites were the most numerous from 10 pm to 3 am, when 57.0% of bites were observed; peak aggressiveness occurred from 11 pm to midnight, with 2.7 BHH. For *An. squamosus*, the HBR was 6.9 BHN, and peak aggressiveness was observed from 8 to 9 pm, with 1.9 bites per hour, 68.5% of which were observed from 6 to 10 pm (Figure 3). In Ankarana, the HBR of *An. funestus* was 1.5 BHN, and no peak was observed during the night. For *An. gambiae* s.s., the HBR was 4.8 BHN; 39.1% of bites of this species occurred from 10 pm to 1 am, and 24.1% occurred from 3 to 6 am. The peak in aggressiveness was observed from 10 to 11 pm, with 0.8 BHH (Figure 3).

#### Sporozoite detection in *Anopheles* spp.

Of the 1,488 anopheline mosquitoes collected via HLC in Amboangisay and Ankarana and tested for human *Plasmodium* sporozoites via ELISA-CSP, 25 (1.7%) were positive for *P. falciparum*, including 24 from Amboangisay and one from Ankarana; none were positive for *P. vivax.* All positive mosquitoes were confirmed to be infected by *P. falciparum* via qPCR testing. Twenty-three (23/25; 92.0%) specimens were collected outdoors. The single sporozoite-positive mosquito in Ankarana was *An. gambiae* s.s. and was also collected outdoors. Of the 24 sporozoite-positive mosquitoes from Amboangisay, 13 (54.2%) were *An. coustani*; all of these specimens were collected outdoors, three of which were collected in the early evening (7–8 pm) and two of which were collected in the early morning (4–5 am). The remaining 11 sporozoite-positive mosquitoes were *An. funestus* (*n* = 4), *An. squamosus* (*n* = 3), *An. gambiae* s.s. (*n* = 2), and *An. mascarensis* (*n* = 2). One of the four sporozoite-positive *An. funestus* mosquitoes were collected indoors between 4 and 5 am.

### Household and individual surveys.

The cross-sectional survey was conducted across 43 households (Table 2). All houses were built of bamboo, wood, or *Ravinala* (palm), and most had holes in the walls through which mosquitoes could enter. In August 2018, 33 (76.7%) households received IRS coverage (18 complete, 15 partial). The number of available ITNs per household ranged from 1 to 5 (median: 2; IQR: 2–3). Twenty-eight (51.2%) households reported an adequate or high number of ITNs, all of which were from the campaign and thus ∼7 months old. All but one household reported washing their ITNs with soap. Thirteen (13/43; 30.2%) households were located within 100 m of a cattle pen. Insecurity was mentioned as an obstacle to seeking care at a health facility by 19 (44.2%) households, and 37 (86.0%) households reported paying for malaria treatment.

**Table 2:** Characteristics of the households surveyed in the communes of Ankarana and Tovona, Farafangana District, Madagascar, 2019

Characteristics	Ankarana (%)	Tovona (%)	Total (%)
Household size: median (interquartile range)	6 (4–8)	8 (6–10)	6 (4–8)
Households with holes in the walls	30 (96.8)	12 (100)	43 (100)
Distance from cattle pens			
≤100 m	10 (32.3)	3 (25.0)	13 (30.2)
ITN coverage			
Inadequate	10 (32.3)	5 (41.7)	15 (34.9)
Adequate	16 (51.6)	6 (50.0)	22 (51.2)
High	5 (16.1)	1 (8.3)	6 (13.9)
ITN washing (water + soap versus water only)			
Water + soap	29 (93.6)	12 (100)	41 (91.3)
IRS coverage			
None	7 (22.6)	3 (25.0)	10 (23.3)
Partial	9 (29.0)	6 (50.0)	15 (34.9)
Complete	15 (48.4)	3 (25.0)	18 (41.9)
Paid for malaria treatment			
Yes	22 (70.9)	9 (75.0)	31 (72.1)
Total	31	12	43

IRS = indoor residual spraying; ITN = insecticide-treated bed net.

Across 43 households, 278 individuals were recorded, and 226 (81.3%) were present and participated in the individual survey (Table 3). Households varied in size, ranging from two to 13 members (median: six). The median participant age was 11 years (IQR: 5–25); 128 (56.6%) participants were female. Nearly all respondents (96%) reported sleeping under an ITN the previous night, and 201 (88.9%) reported consistent use of ITNs. Seventeen individuals reported a reason for inconsistent ITN use, 13 (76.5%) of whom stated that they slept outdoors to protect their property; the remaining individuals reported either insufficient ITNs or being away from home.

**Table 3:** Characteristics of surveyed individuals in the communes of Ankarana and Tovona, Farafangana District, 2019

Characteristics	Ankarana	Tovona	Total
Sex (% males)	66 (44.9)	32 (40.5)	98 (43.4)
Age (years)			
0–5	44 (29.9)	24 (30.4)	68 (30.1)
6–14	39 (26.5)	26 (32.9)	65 (28.8)
15–49	51 (34.7)	23 (29.1)	74 (32.7)
>49	13 (8.8)	6 (7.6)	19 (8.4)
Current/recent fever	18 (12.2)	2 (2.5)	20 (8.8)
Fever in previous 2 weeks	22 (15.1)	15 (19.2)	37 (16.4)
Distance from cattle pens			
≤100 m	49 (33.3)	23 (29.1)	72 (31.9)
ITN use the previous night	140 (95.2)	77 (97.5)	217 (96.0)
ITN use the previous 2 weeks			
Nightly	129 (87.8)	72 (91.1)	201 (88.9)
Irregularly	15 (10.2)	6 (7.6)	21 (9.3)
Never	3 (2.0)	1 (1.3)	4 (1.8)
IRS exposure	113 (76.9)	55 (69.6)	168 (74.3)
Total	147	79	226

IRS = indoor residual spraying; ITN = insecticide-treated bed net.

### Care-seeking behavior and malaria prevention practices.

Of 226 respondents, 37 (16.4%) reported having a fever in the previous 2 weeks (Table 3). Of these, 23 (62.2%) were female, and the median age was 9 years (IQR: 4–26); 19 (19/37; 51.4%) respondents sought no care or self-medicated, whereas 14 (14/37; 37.8%) sought care from a CHV, and four (10.8%) sought care at a health facility. Of the 18 (18/37; 48.6%) who sought care, 15 (83.3%) underwent an RDT, 13 (86.7%) of which were positive. Among nine women who reported being pregnant during the previous year, six (66.7%) had attended at least one antenatal consultation, and one (11.1%) reported taking at least three preventative treatments with sulfadoxine–pyrimethamine (SP).

### Malaria test results.

Of 226 individuals who underwent RDTs, 71 (31.4%) were positive, 61 (85.9%) of whom were asymptomatic (Table 4). At least one RDT-positive individual was identified in each of the 32 (74.4%) households. Microscopy results were available for 61 individuals with positive RDT results; of these, *P. falciparum* was the predominant species (43/61; 70.5%). Of the remaining 18, one had *Plasmodium ovale*, and 17 had negative microscopy results. The age group with the highest infection rate was 6–14 years old.

**Table 4:** Characteristics associated with confirmed malaria cases, Farafangana District, April 2019

Characteristics	Total, *N* (%)	RDT (+), *n* (%)	OR (95% CI)	*P*
Age (years)				
0–5	68 (30.1)	19 (26.8)	1	
6–14	65 (28.8)	31 (43.7)	2.4 (1.1–4.8)	0.02
15–49	74 (32.7)	18 (25.3)	0.8 (0.4–1.8)	0.6
>49	19 (8.4)	3 (4.2)	0.5 (0.2–0.7)	0.3
Current/recent fever	20 (8.8)	10 (14.1)	2.4 (0.9–6.0)	0.1
Sex				
Female	128 (56.6)	36 (50.7)	1	
Male	98 (43.4)	35 (49.3)	1.4 (0.8–2.5)	0.2
Distance from cattle pens				
≥100 m	154 (68.1)	50 (70.4)	1	
<100 m	72 (31.9)	21 (29.6)	0.9 (0.5–1.6)	0.6
ITN use in the previous 2 weeks				
Never or irregularly	25 (11.1)	10 (14.1)	1	
Nightly	201 (88.9)	61 (85.9)	0.7 (0.3–1.5)	0.3
ITN use the previous night				
No	9 (4.0)	5 (7.0)	1	
Yes	217 (96.0)	66 (93.0)	0.3 (0.1–1.3)	0.1
IRS coverage				
None	58 (25.7)	20 (28.2)	1	
Partial or total	168 (74.3)	51 (71.8)	0.8 (0.4–1.6)	0.6
Sample size	226	71	–	–

IRS = indoor residual spraying; ITN = insecticide-treated bed net; OR = odds ratio; RDT = rapid diagnostic test.

## DISCUSSION AND CONCLUSION

In the current investigation, the aim was to identify the drivers of focal malaria outbreaks in the Farafangana District, Madagascar, in 2018. The present study revealed that outdoor biting vectors played an important role in increasing and sustaining parasite transmission. *Anopheles coustani*, a secondary vector that was the predominant species detected during the entomological investigation, exhibited high sporozoite positivity, indicating its contribution to malaria transmission. Few vectors were captured indoors, suggesting that the recent IRS and ITN distribution campaigns, although likely effective against indoor biting vectors, provided little or no protection against confirmed vectors with outdoor feeding behavior. Furthermore, the household survey conducted in affected communities revealed considerable asymptomatic infections, which likely contributed to ongoing residual transmission. Individuals with asymptomatic infection may spend more time outdoors compared with those who are symptomatic and would be less likely to seek care or treatment. Although variations in malaria prevalence and focal malaria outbreaks are not uncommon in this area, nor are asymptomatic infections.^2^^,^^17^ The present investigation highlighted the risk posed by outdoor-biting vectors. This risk is particularly pronounced when asymptomatic infections, which often go undetected and untreated, are widespread.^18^

*Anopheles coustani* was first identified as a malaria vector in Madagascar in 2013 during an entomological investigation in the Central Highlands, where it was found to be the predominant vector species.^19^ Other studies have since revealed that *An. coustani* is an important malaria vector in Madagascar. For example, in the Maevatanana and Ifanadiana Districts, which border Farafangana, both *P. falciparum* and *P. vivax* were detected in *An. coustani.*^20^^,^^21^ However, the present investigation was the first to include both entomological and epidemiological data supporting transmission. Nearly all *P. falciparum* sporozoite-positive mosquitoes were collected outdoors, and all mosquito species except *An. funestus* exhibited outdoor biting behavior. In addition, it is noteworthy that *An. coustani*’s peak biting occurred during the early evening or early morning hours, when many individuals, including older children, tend to be outdoors for socializing or work.

The ITNs distributed in Farafangana in 2018 contained the pyrethroid insecticide deltamethrin. Baseline measures of insecticide efficacy on the ITNs revealed that the mosquito knockdown rate ranged from 66% to 67%, which is below the WHO-recommended threshold of 95%. Additionally, the 24-hour mosquito mortality rate ranged from 84% to 85% which falls at the WHO threshold (80%; IPM, unpublished data). The insecticide susceptibility of vectors was not measured during the present investigation. However, testing performed in 2015 and 2016 in a village in the Farafangana District revealed that *An. gambiae* s.l. was fully susceptible to bendiocarb, dichloro-diphenyl-trichloroethane, deltamethrin, lambda-cyhalothrin, permethrin, and pirimiphos-methyl using WHO tube tests. *Anopheles gambiae* s.l. was also susceptible to these insecticides, including alphacypermethrin, as indicated by the CDC bottle bioassay testing conducted at that time. *Anopheles funestus* s.l. specimens were susceptible to pirimiphos-methyl and deltamethrin, whereas *An. mascarensis* was susceptible to deltamethrin, as indicated by CDC bottle bioassay tests. No testing was performed on *An. coustani* specimens.^22^ Although it is possible that insecticide resistance increased between 2015 and 2018, the fact that most households in this survey reported washing ITNs with soap may have affected the insecticide efficacy. Nonetheless, the ITNs distributed in 2018 should still have offered barrier protection against biting. Routine post-IRS testing suggested that residual insecticide from the IRS campaign in August 2018 would remain effective until April 2019.^10^ The lack of evidence that ITNs or IRS were ineffective, combined with entomological evidence demonstrating outdoor biting of sporozoite-positive vectors, suggests that transmission was due to exophagic vectors rather than a failure of vector control tools (ITN and IRS).

Household and individual surveys revealed that most RDT-confirmed infections were asymptomatic, and nearly half of all infections occurred among children aged 6–14 years. Infections among children in this age group are well-described in Madagascar, and they are more likely to be asymptomatic than those among younger children.^3^^,^^17^^,^^23^ Other studies have revealed that older children are more likely to be outdoors later in the evening for socializing or work than their younger counterparts.^24^ Given the outdoor biting with sporozoite-positive vectors, this could explain the high infection rates in this age group. Nearly all survey respondents reported the consistent use of ITNs, although more than 30% noted an inadequate number of ITNs in the household. This discrepancy suggests that ITN use may have been over-reported. Of those who did not report consistent ITN use, most stated that it was due to sleeping outdoors, which would increase their risk of exposure to infectious mosquito bites. Proximity to cattle pens did not increase the risk of malaria in the present study. However, it has been reported as a risk in other studies, and the role that herds play in sustaining transmission in cattle-raising areas of Madagascar remains unclear.^21^^,^^25^

The proportion of study respondents who reported fever within the previous 2 weeks (16.2%) was higher than in surveys conducted more recently.^17^^,^^26^ The proportion of asymptomatic infection was highest among respondents aged >15 years (>95%); however, even among children aged 0–5 years and 6–14 years, most infections were asymptomatic (82% and 84%, respectively). Asymptomatic infections have been reported in this area of Madagascar^17^^,^^27^ and are likely to have contributed to sustained transmission, as has been described elsewhere.^28^

More than half of the respondents for this survey indicated that they did not seek care for fever, which is consistent with results reported in other studies.^17^ Among the nine women who reported a pregnancy in the previous year, only one received at least three preventative doses of SP as recommended; no additional questions regarding antenatal care (ANC) were assessed. Barriers to care-seeking for fever and ANC in Madagascar include distance to health facilities, concerns about facility closures and commodity stockouts, perceptions of poor-quality care, and cultural beliefs.^29^ These factors likely also contributed to limited care-seeking during the outbreak. Fees charged for malaria testing and treatment have also been described as a barrier.^29^ In the present investigation, 59% of respondents indicated that they paid for malaria treatment. Madagascar has been exploring methods for reducing barriers to care-seeking, including policies to allow CHVs in remote areas to test and treat all community members for malaria and implement proactive community case management. A 2021 study on extending malaria community case management to all ages in Farafangana, which included adequate RDTs and ACTs, as well as remuneration of CHVs, increased care-seeking in both intervention and control villages.^30^ A study conducted in a neighboring district revealed that biweekly home visits performed by CHVs to test individuals with fever also increased prompt malaria testing and treatment. These findings relied on ensuring a consistent and reliable supply of RDTs and ACTs at the CHV level.^21^ Stock supplies were not assessed in the present study; however, it is possible that these may have been lacking in sufficient quantity.

This investigation had limitations. Primarily, there was a delay between outbreak detection and the initiation of the investigation. Village access was difficult because of washed-out roads and security concerns, and entomological and epidemiological investigations were not conducted simultaneously or in the same villages. A distance of 10 km and a delay of 2 months could reflect substantial variability in vector and parasite activity. In field sites investigated for the entomological survey, outdoor mosquito collection and breeding site identification were challenging because of seasonal rains that limited vector data. In addition, mosquito collections were performed at night (6 pm–6 am), and daytime biting might have been missed. Indeed, the HBR of *An. coustani* was high at both 6 am and 6 pm (2.5), which suggests that its biting activity might last later into the morning and begin earlier in the evening, as has been described in Central Africa.^31^ Finally, health system data were not collected; therefore, factors such as supply stockouts, which may have contributed to the outbreak, could not be determined.

Since the completion of the present investigation, the estimated prevalence of malaria in the Atsimo-Atsinanana Region has continued to increase,^3^ and efforts to improve access to care are being scaled up.^27^^,^^30^ However, in settings where malaria vectors exhibit outdoor biting behavior and malaria infections are asymptomatic or untreated, interventions aimed at preventing indoor biting will be inadequate for preventing transmission. Initiatives aimed at addressing the risks of outdoor biting and reducing barriers to care-seeking should be considered. In addition, determining the daytime outdoor biting behavior of vectors could provide valuable insights. Finally, supporting local public health authorities in detecting malaria clusters and conducting rapid, systematic investigations could improve response quality.

## Supplemental Materials

10.4269/ajtmh.24-0834 Supplemental Materials
