# Supplementary material for: Malaria Outbreak in Farafangana District, Southeast Madagascar, 2018: Are Secondary Vectors a Threat to Current Malaria Control Approaches?
Source: Am J Trop Med Hyg. 2025 Oct 23;114(2 Suppl):52–60. doi: 10.4269/ajtmh.24-0834 (PMC12874837; doi:10.4269/ajtmh.24-0834)
Supplement: Supplemental Materials [file tpmd240834.SD1.pdf]

Supplementary Table 1

Description of variables collected

Variables	Definition
Current fever	Axillary temperature ≥ 37.5°C at the time of the survey
Recent fever	Self-report of fever within previous 48 hours
Confirmed malaria cases	Person with positive malaria rapid diagnostic test
Symptomatic malaria cases	Person with positive malaria rapid diagnostic test with current or recent fever
Asymptomatic malaria cases	Person with positive malaria rapid diagnostic test without current or recent fever
Indoor residual spraying (IRS) coverage	None<br>Partial: at least one room used for sleeping was not sprayed<br>Complete: Every room used for sleeping was sprayed
Insecticide-treated bed net (ITN) coverage	Inedaquat : less than one ITN for every three individuals<br>Adequate: At least one ITN for every three individuals<br>High: More than on ITN for every three individuals
